# Molecular Mechanisms of DNA Sequence Selectivity in V(D)J Recombination

**DOI:** 10.1021/acsomega.3c05601

**Published:** 2023-09-15

**Authors:** Walker Hoolehan, Justin C. Harris, Karla K. Rodgers

**Affiliations:** ^†^Department of Biochemistry and Molecular Biology, ^§^Department of Microbiology and Immunology, University of Oklahoma Health Sciences Center, Oklahoma City, Oklahoma 73104, United States

## Abstract

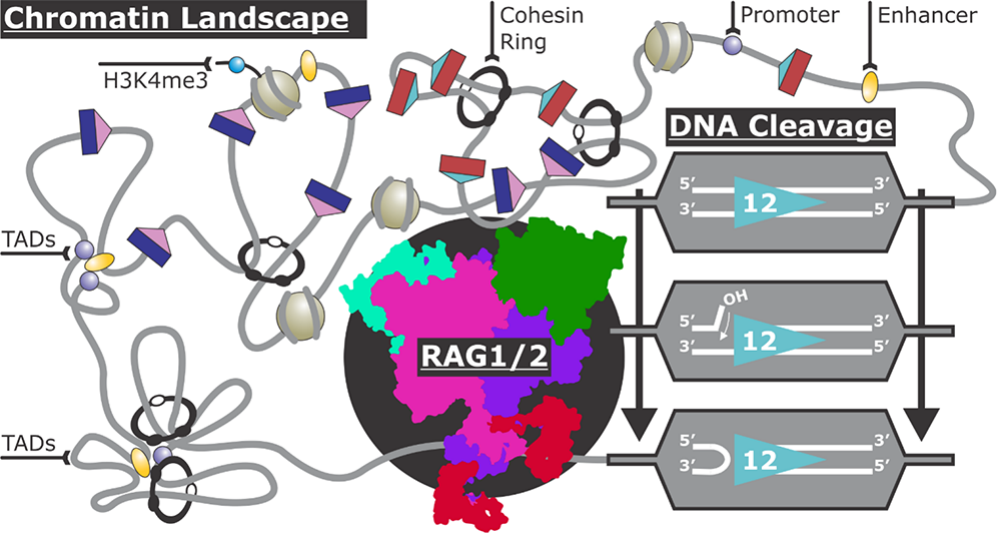

Antigen receptor (AgR) diversity is central to the ability of adaptive immunity in jawed vertebrates to protect against pathogenic agents. The production of highly diverse AgR repertoires is initiated during B and T cell lymphopoiesis by V(D)J recombination, which assembles the receptor genes from component gene segments in a cut-and-paste recombination reaction. Recombination activating proteins, RAG1 and RAG2 (RAG1/2), catalyze V(D)J recombination by cleaving adjacent to recombination signal sequences (RSSs) that flank AgR gene segments. Previous studies defined the consensus RSS as containing conserved heptamer and nonamer sequences separated by a less conserved 12 or 23 base-pair spacer sequence. However, many RSSs deviate from the consensus sequence, and the molecular mechanism for semiselective V(D)J recombination specificity is unknown. The modulation of chromatin structure during V(D)J recombination is essential in the formation of diverse AgRs in adaptive immunity while also reducing the likelihood for off-target recombination events that can result in chromosomal aberrations and genomic instability. Here we review what is presently known regarding mechanisms that facilitate assembly of RAG1/2 with RSSs, the ensuing conformational changes required for DNA cleavage activity, and how the readout of the RSS sequence affects reaction efficiency.

## V(D)J Recombination Overview

**Figure 1:**
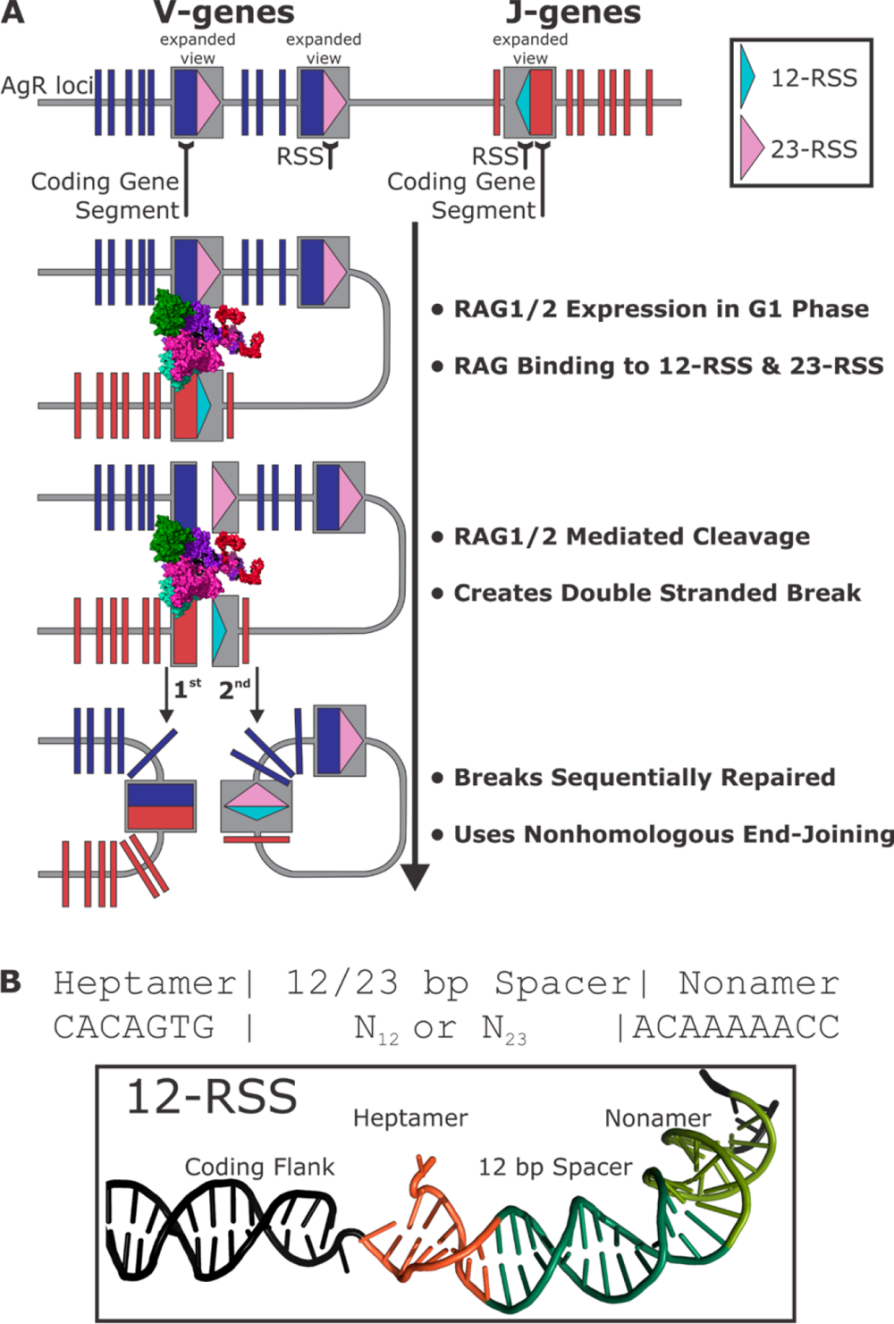
(A) Schematic representation of a simplified AgR light chain locus undergoing V(D)J recombination. With upstream, dark-blue V-gene segments flanked by pink 23-RSSs, and downstream red J-genes flanked by light blue 12-RSSs. The V-genes, J-genes, and accompanying RSSs are brought together by a multilayered mechanism. RAG1/2 recognizes and binds to 12-RSS and 23-RSS. RAG1/2 proceeds to cleave between the RSSs and the gene segments, creating two DSBs at both bound RSSs. Coding-ends are first joined by NHEJ to create a coding joint, and then, RSSs are joined by NHEJ machinery, creating a signal joint on an excised circular DNA fragment. (B) Cryo-EM structure of the nicked 12-RSS (from PDB: 6CIJ). The coding flank is in black. The 12-RSS is colored by conserved motifs: orange = heptamer, teal = spacer, and green = nonamer.

The power of adaptive immunity stems from the diverse sequences of antigen receptor (AgR) genes expressed by the ensemble of B- and T-cells in the immune system of gnathostomes (jawed vertebrates).^1^ The combined AgR repertoires contain a vast array of binding specificities, resulting in the ability to specifically bind and target virtually any antigen. From a limited amount of genetic material, V(D)J recombination initiates the production of the AgR repertoires during B- and T-cell development through differential rearrangement of V (variable) and J (joining) gene segments (Figure 1A).^1^ In some AgR loci, D (diversity) gene segments are also present, requiring two consecutive recombination events (D-J followed by V-D-J) to generate a functional AgR gene. Both B- and T-cells share the same molecular machinery for performing V(D)J recombination, but generally, immunoglobulin (Ig) loci are rearranged in B-cells, while T-cell receptor (TCR) loci are rearranged in T-cells. Each AgR gene locus is uniquely arranged, and different species of jawed vertebrates can have different arrangements for each AgR gene locus. In the germline orientation of mammalian AgR loci, the V gene segments are typically dispersed over a large region that is up to 2 megabases in length and are separated from the other classes of gene segments. After productive rearrangements, Ig heavy (IgH) and light chain (Igκ or Igλ) proteins in B-cells and TCR α and β chains in T-cells form heterooligomeric receptor complexes at the cell surface.

B-cells develop in the bone marrow where the IgH gene locus is first recombined when a D gene is joined to a J gene, and then a V gene is joined to the prerearranged DJ gene. After productive VDJ rearrangement, the heavy chain forms a pre-B Cell Receptor (pre-BCR) on the cell surface by pairing with an invariant light chain. AgR gene rearrangements typically occur on only one allele, and enforcement of this phenomenon is known as allelic exclusion. If the pre-BCR passes positive/negative selection, then the Ig light chain (Igκ or Igλ) gene locus is recombined. If initial light-chain gene rearrangements produce an autoreactive BCR, the light chain gene locus may undergo receptor editing, wherein RAG1/2 performs a second V(D)J rearrangement with a new VJ gene pair. After productive rearrangement and formation of a nonautoreactive BCR, B-cells emigrate from the bone marrow into the periphery. B-cells expressing a membrane-bound BCR are called naïve B-cells until they encounter antigen, at which point they can undergo somatic hypermutation and class switching to further enhance specificity to the antigen, followed by differentiation to memory cells or antibody-producing plasma cells.

While B-cell development occurs in the bone marrow, T-cells that express αβ or γδ TCRs develop in the thymus. The development of αβ TCR-expressing T-cells will be discussed here, as these cells represent the vast majority of T-cells in the adaptive immune system. In early T-cell development, the TCRβ locus is first recombined when a D gene is joined to a J gene, and then a V gene is joined to the prerearranged DJ gene. After a productive VDJ rearrangement, the TCRβ chain forms a pre-TCR on the cell surface by pairing with an invariant light chain. If the pre-TCR passes positive selection, then the TCRα gene locus is recombined. TCRα gene rearrangement is not subjected to allelic exclusion, and a significant percentage of T-cells express dual αβ TCRs. After productive rearrangement of α/β genes that are not autoreactive, T-cells commit to CD4 or CD8 lineage and emigrate from the thymus as naïve T-cells. Altogether, lymphocyte development ensures sufficient AgR diversity to neutralize myriad pathogens while preventing deleterious, autoreactive lymphocytes from escaping the primary lymphoid organs.

## RAGs

The molecular mechanism of V(D)J recombination is conserved across all gnathostomes.^1^ The recombination activating proteins, RAG1 and RAG2, are both required to initiate V(D)J recombination by catalyzing site-specific DNA cleavage reactions at the border of selected AgR gene segments. All of the gene segments are flanked by semiconserved DNA sequences, referred to as recombination signal sequences (RSSs), which target the RAG proteins to appropriate DNA cleavage sites (Figure 1B). Each RSS consists of heptamer and nonamer sequence elements separated by a poorly conserved spacer of either 12 or 23 bp (12-RSS and 23-RSS). One of each type of RSS is required for a V(D)J recombination event, which is known as the 12/23 rule.^1^ The required asymmetry in the RSS spacer length for V(D)J recombination activity helps direct the proper order of recombination events. For example, in mouse and human Igκ loci, all V and J genes are flanked by12-RSSs and 23-RSSs, respectively. This arrangement permits V-to-J joining but prevents V-to-V or J-to-J joining events. An exception to this arrangement is in the TCRβ locus, where V-to-J recombination could occur, as they are flanked by RSSs of differing spacer length. However, the occurrence of D-less TCRβ is of low frequency and its restriction is referred to as the “beyond 12/23 rule”.^1^

**Figure 2:**
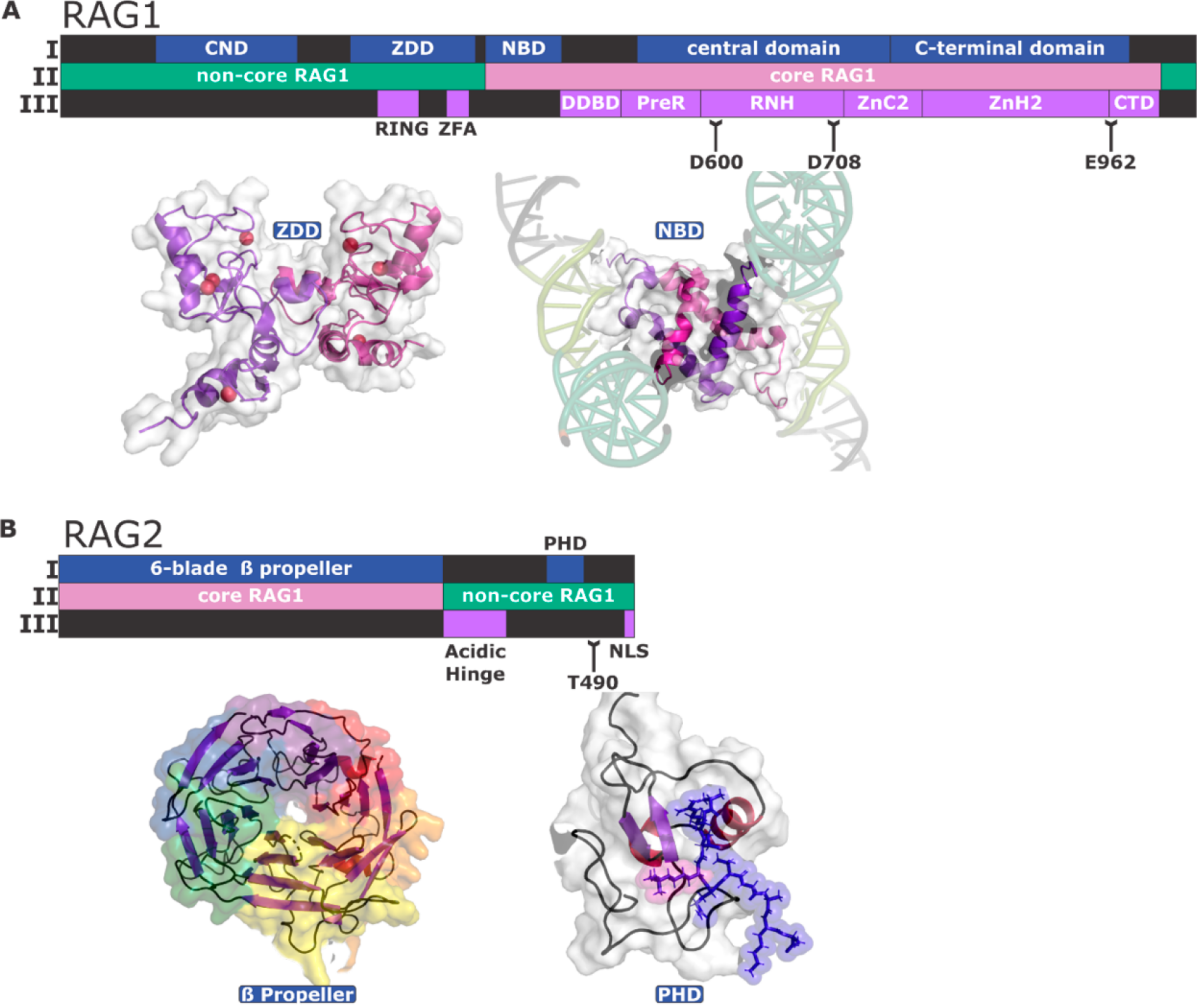
(A) Diagram of mouse RAG1 domains. (I) The topologically independent domains of RAG1 are shown in dark blue. These include the central noncore domain, CND; zinc dimerization domain, ZDD; nonamer binding domain, NBD; central domain; and C-terminal domain. (II) Core RAG1 regions are shown in dark pink (389–1008 aa) and noncore RAG1 (residues 1–388 and 1009–1040) regions in dark teal. (III) Functional domains of RAG1 are shown in light purple. These include the RING finger domain, RING; Zinc Finger A domain, ZFA; Dimerization and DNA binding domain, DDBD; pre-RNase A domain, PreR; Catalytic RNase H, RNH; Zinc binding and coordinating domains, ZnC2 and ZnH2; C-terminal domain, CTD. The DDE active site residues are shown in black below the RAG1 bar diagram. The ribbon diagram of the ZDD (PDB: 1RMD) is shown below the RAG1 bar diagram (at left). The ZDD subunits are pink or purple, with zinc ions shown as red spheres. The NBD (PDB: 3JBW) is shown to the right. Each NBD subunit is colored pink or purple and is shown in complex with the RSS nonamer (shown in light gray). (B) Diagram of mouse RAG2 domains. (I) Topologically independent domains of RAG2 are shown in dark blue. These include the 6-blade ß propeller and the Plant Homeodomain, PHD. (II) Core (residues 1–352) and noncore regions of RAG2 (residues 353–527) are shown in dark pink and dark teal, respectively. (III) Functional domains of RAG2 are shown in light purple. These include the Acidic Hinge and the Nuclear Localization Signal, NLS. Residue T490 is shown in black below the RAG2 bar diagram. The ribbon diagram of the 6-bladed ß propeller structure of core RAG2 (PDB: 4WWX) is shown at left below the RAG2 bar diagram. Each blade’s surface is colored red, orange, yellow, green, blue, and purple. To the right is the ribbon diagram of RAG2’s PHD in complex with an H3K4me3 peptide (PDB: 2V89). The H3 peptide backbone and side chains are shown in a stick depiction with transparent spheres overlaid. The trimethylated lysine 4 residue is highlighted with pink spheres.

Multiple laboratories began biochemically characterizing the RAG:RSS interaction to determine how RAG1/2 interacts with RSSs to facilitate V(D)J recombination (reviewed in ref (1)). The core catalytic domains of RAG1/2 were discovered (Figure 2), and the expression of affinity-tagged core RAG1/2 (cRAG1/2) proteins enabled purification of sufficient quantities of protein for *in vitro* biochemical assays. Importantly, the precise definition of “core” RAG1/2 has been refined since the initial discovery of dispensable noncore protein domains three decades ago. More recently, the definition of cRAG1/2 was aligned with structural domains determined by recently solved murine cRAG1/2 structures (reviewed in ref (2)), where amino acid (aa) residues 384–1008 (out of 1040 total) form the cRAG1 catalytic domain and aa residues 1–352 (out of 527 total) form the cRAG2 beta propeller domain (Figure 2B).

cRAG1 contains the DNA cleavage active site, termed the DDE motif, which consists of conserved acidic residues that coordinate two divalent metal cations in the active site (Figure 2A).^1,3^ Divalent cations facilitate nucleophilic attack by activating a water molecule and by coordinating a 3′-OH group for the nicking and hairpin formation steps, respectively. In addition, cRAG1 contains the DNA binding domains that are specific for the RSS heptamer and nonamer. cRAG2, which consists of a six-bladed propellor structure, is essential for DNA cleavage activity and increases the DNA sequence specificity of RAG1 for the RSS.

The noncore regions regulate RAG protein localization, degradation, and chromatin targeting.^1,3^ To date, high-resolution structures are known for the core regions of both RAG proteins bound to 12- and 23-RSSs, but the orientations of the noncore RAG regions within the RAG:RSS complexes are not known. The noncore RAG regions are important for complete activity in developing lymphocytes. For example, cRAG2 knock-in mice show a profound lack of B- and T-cells, as full length RAG2 is crucial for long-range recombination events, such as V-to-DJ joining, in pro-B and pro-T cells. It was later determined that noncore RAG2 binds specifically to the trimethylated K4 residue in Histone H3 (H3K4me3).^4^ This interaction is mediated through the RAG2 plant homeodomain (PHD), a zinc-binding domain that spans residues 446–481 (Figure 2B). While this interaction provides a docking site for RAG2 (and through association, RAG2-bound RAG1) to AgR loci that are enriched with the H3K4me3 modification, it also recruits RAG1/2 complexes to multiple open chromatin sites, particularly transcription start sites (TSSs) throughout the genome, as reported by ChIP-seq experiments.^4^

Accumulating evidence supports the hypothesis that gnathostome RAG1/2 evolved from a RAG-like transposase. RAG1/2’s nick-hairpin DNA cleavage mechanism is also shared among several transposase families, along with the recent identification of RAG1/2-like transposases in invertebrates.^5^ Importantly, the full-length gnathostome RAG1/2 complex lacks transposase activity because the strand transfer step is inhibited by the noncore region of RAG2.^5^

Mutations of the *RAG* genes in humans lead to a wide range of primary immunodeficiency diseases (PIDs) that are associated with a broad spectrum of clinical phenotypes.^6^ Complete defects in RAG expression or activity result in severe combined immunodeficiency (SCID), which presents clinically within the first few months of life. However, hypomorphic RAG mutations can result in a milder impairment of adaptive immunity that manifests as combined immune deficiency (CID), which is often associated with granulomas and/or autoimmunity (CID-G/AI). These latter conditions typically have a delayed clinical onset of several years that can lead to increased challenges toward successful treatments.

## The V(D)J Recombination Mechanism

**Figure 3:**
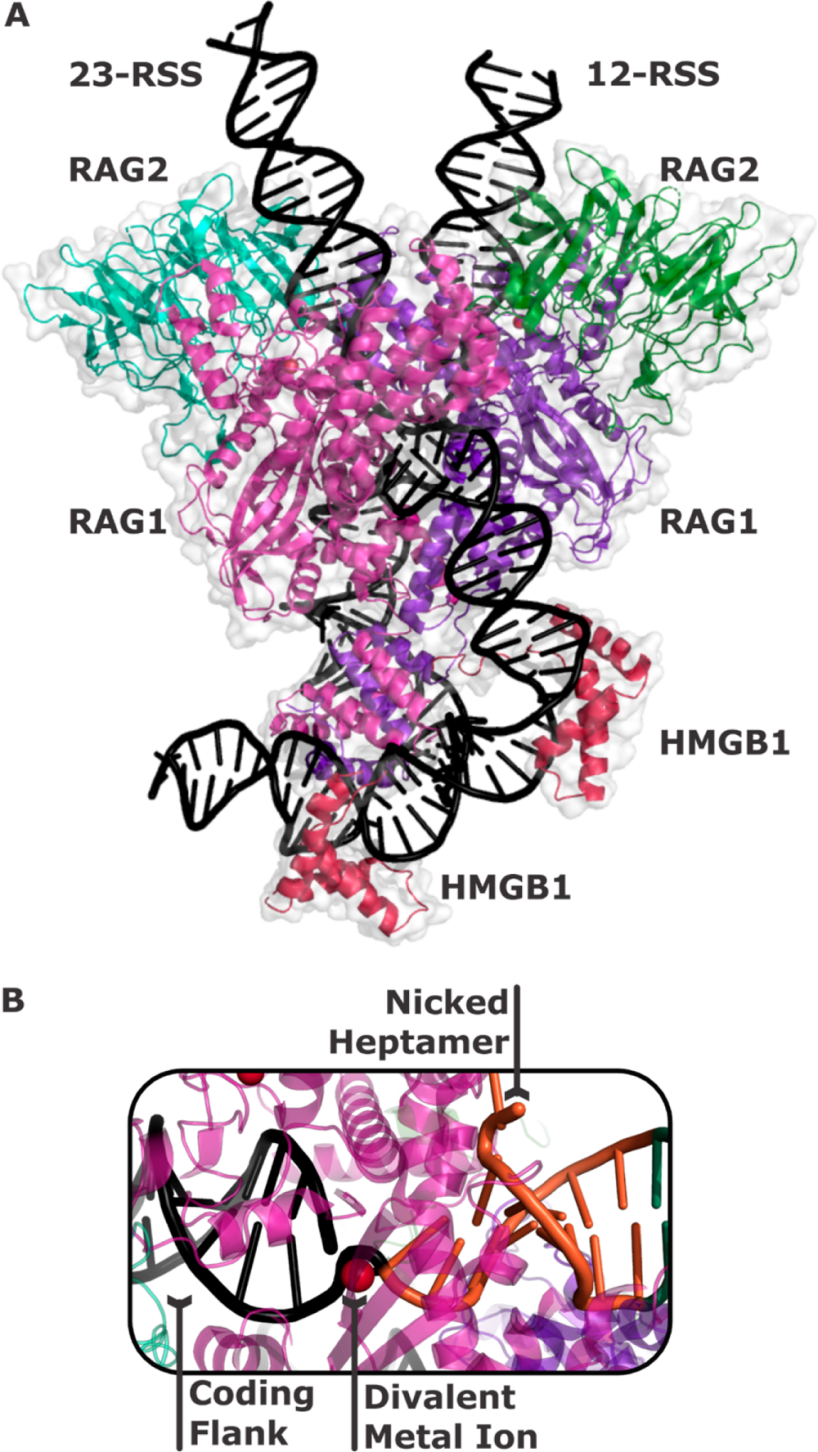
(A) A cartoon model of the RAG1/2 heterotetramer (PDB: 6CIJ) bound to a 12- and 23-RSS. The two RAG1 subunits are in pink and purple, and the two RAG2 subunits are colored teal and green. Both RSSs are shown in black. Partial structures for HMGB1, bound to the 23-RSS spacer, are shown in red. (B) A zoomed-in view of the nicked 23-RSS in the active site of RAG1 (PDB: 6CIJ). The RAG1 protein is colored in panel A, and the 23-RSS heptamer and coding flank are colored orange and black, respectively. A divalent cation bound in the active site is represented by a red sphere.

During V(D)J recombination, the RAG proteins form structurally distinct complexes with the 12/23-RSS pair during a two-step DNA cleavage reaction, as illustrated by high-resolution cryo-EM and crystal structures (example shown in Figure 3).^2,7^ First, the heterotetrameric RAG1/2 complex binds a 12- and a 23-RSS to form a paired complex (PC). Formation of the PC requires the high mobility group protein HMGB1 or HMGB2. In cryo-EM structures of RAG1/2 complexed with a consensus RSS pair, HMGB1 was found bound to the 23-RSS spacer (Figure 3).

RAG1 nicks the phosphodiester linkage between the RSS heptamer and the adjacent coding flank. Divalent metal ions, coordinated by the DDE amino acid side-chains in the RAG1 active site, activate a water molecule to facilitate nucleophilic attack of the phosphorus atom, liberating a 3′-OH at the coding end. Nicking is accompanied by conformational changes that include base-flipping at the heptamer/coding flank border and places the 3′OH in an optimal position to attack the phosphodiester linkage of the opposite strand, which yields a blunt double-strand break (DSB) at the signal ends and hairpin-sealed coding ends (Figure 1A). Hairpin formation at the coding ends is a coupled reaction that requires both RSSs and is the basis of the 12/23 rule. That DNA cleavage requires binding of the heterotetrameric RAG1/2 complex to both RSSs is a shared feature with other nucleic acid-cleaving enzymes. For example, certain subtypes of Type II restriction endonucleases require binding to two DNA substrates for coupled cleavage at both recognition sites.^8^ Structures of the RAG1/2 PC reveal that each RAG1 subunit simultaneously bridges both RSS heptamers, allowing communication between the active sites of the heterotetrameric complex that likely enforces coupled cleavage.

The RAG complex remains bound to the coding and the RSS signal ends in a postcleavage complex, with release of the coding ends first to nonhomologous end joining (NHEJ) factors. Hairpin-sealed coding ends are nicked by Artemis, terminal transferase (TdT) adds additional bases to the junction to further enhance diversity, and NHEJ machinery joins the coding ends together to form a coding joint.^9^ Two consecutive recombination events in AgR loci containing D segments or one event in loci without D segments yield the exon that, if in-frame, codes for the antigen binding region of the AgR (Figure 1A). Developing lymphoid progenitors may undergo additional rounds of recombination to produce a functional, in-frame AgR chain. In a functional AgR chain, the highly variable coding joints code for the CDR3, one of the hypervariable loops of the AgR that determine B- and T-cell specificity by forming the primary AgR:antigen surface contacts. RAG1/2 ultimately dissociates from the signal ends, and the NHEJ machinery typically joins the ends precisely to repair the DSB (Figure 1A). In most cases, RAG1/2 cleaves convergently oriented RSS pairs, which deletes large segments of DNA intervening the coding ends, and subsequent signal end joining forms an extrachromosomal circular DNA fragment.^1^ Otherwise, inversional recombination events occur between identically oriented (forward–forward or reverse–reverse) RSSs where both the coding and signal joints are retained in the chromosome.^1^

## RSS

RAG1/2 specificity for recombination signal sequences (RSS) is central to the V(D)J recombination paradigm. Over four decades ago, AgR gene sequencing results showed the presence of semiconserved RSSs at the border of each of the V, D, and J gene segments. Over time, as more AgR gene loci were sequenced, the definition of the RSS was refined to the consensus CACAGTG-N_12/23_-ACAAAAACC sequence, which is still accepted as the canonical RSS. However, many RSSs do not contain the canonical consensus motif, sometimes substantially deviating from the canonically defined consensus heptamer and nonamer sequences. Because the RSSs are only somewhat conserved, RAG1/2 must recombine divergent RSSs to realize the complete AgR repertoire. However, cryptic RSS-like sequences (cRSS) are also present throughout the genome.^1^ RAG1/2-mediated cleavage at cRSSs can lead to chromosomal aberrations, such as oncogenic translocations.^10^ Overall, RAG1/2 specificity must be promiscuous for complete realization of the AgR repertoire while simultaneously avoiding deleterious rearrangements of cRSSs, and in this review, we discuss present models and future directions for better understanding V(D)J recombination specificity and its biological implications.

## Biochemical Studies of RAG:RSS Complex Formation and Cleavage Mechanism

Following the discovery of the RAG1/2 proteins, researchers sought to determine the biological significance of the protein complex recognized by various RSSs. Biochemical studies of RAG1/2 specificity included in vitro DNA binding and cleavage assays, as well as extrachromosomal V(D)J recombination assays.^1^ These combined studies confirmed that the canonical consensus RSS was a preferred substrate for RAG1/2. The first three heptamer bases, CAC, were consistently required for appreciable RAG-mediated cleavage. Neither the consensus nonamer nor consensus heptamer positions 4–7 were absolutely required for cleavage, but the presence of both significantly enhanced RAG1/2 cleavage activity. The coding-flank sequence also impacted RAG1/2 activity with differential DNA cleavage activity spanning orders of magnitude. Various spacer sequences were also extensively assayed using extrachromosomal recombination assays.^11^ A 5′-AT-3′ “consensus” spacer motif was preferred directly adjacent to the RSS heptamer. Heptamer specificity is partly determined by RAG1 contacts at heptamer positions 5–7. Cryo-EM and X-ray crystallographic studies revealed RAG1 contacts at heptamer positions 5–7 through the minor groove and base-specific thymine contacts at heptamer position 6.^12^ Chemical probe assays confirm RAG-mediated steric effects at heptamer positions 5–7 in solution.^13^

Biochemical DNA cleavage assays with purified RAG1/2 and 12/23-RSS-containing oligonucleotide duplexes first demonstrated that DNA double-strand breaks at a 12- and 23-RSS were generated in a coupled cleavage manner in the context of the PC.^1^ Coupled cleavage events occurred only in the presence of Mg^2+^ and HMGB1/B2. In contrast, DNA cleavage was uncoupled, with nicking and hairpin formation occurring at a single RSS in Mn^2+^-containing buffers. Finally, the addition of Ca^2+^ rendered the RAG1/2 enzyme complex inactive, although it was capable of forming the PC. These divalent metal requirements are similar to that observed with other nucleic-acid cleaving enzymes. More advanced single-molecule (sm) studies have provided detailed kinetic parameters for RAG1/2 binding and cleavage.^14−16^ For example, sm colocalization studies confirmed that the RAG1/2 dwell time on DNA substrates is substantially longer upon PC formation, while smFRET demonstrated the extent of DNA bending in the RSSs in the PC.^14^ Also, sm tethered particle motion assays tested RAG1/2 binding and cleavage activity on dozens of RSSs, which showed that RAG:RSS binding and cleavage kinetics are differentially affected by various heptamer and nonamer mutations.

Since assaying RAG activity on variant RSSs one at a time is time-consuming and labor-intensive, most studies focused on characterizing variant RSSs that are implicated in physiological processes such as *bona fide* RSS rearrangement, oncogenic translocation at cRSSs, and/or V-gene replacement using cRSSs. However, these studies were often performed in different experimental contexts, making it difficult to draw accurate comparisons of the RAG1/2 specificity between different studies. Until very recently, RAG1/2 activity on variant RSSs was ill-defined, and because many active RSSs lack canonical consensus heptamer/nonamer motifs, a more accurate, comprehensive understanding of RAG:RSS specificity was needed to accurately classify putative RSSs and cRSSs.

## RIC

The quality of RSSs is often predicted by an RSS information content (RIC) score, a statistical model derived to predict RSS efficacy based on DNA sequence alone.^17^ RIC scores helped identify cRSSs that were apparently cleaved by RAG1/2; however, while RIC was useful for identifying sequences containing conserved RSS elements, recent studies have noted poor correlation between RSS/cRSS recombination frequencies and RIC scores.^18^ Discrepancies between predicted RSS efficacy and true RSS efficacy could partly be a result of RIC scores being derived from specific trends in the conservation of RSSs, which is not necessarily driven by the maximization of RAG1/2 activity. Specifically, the quality of RSSs may have evolved to modulate the RAG1/2 activity at certain locations within AgR loci. For example, although highly utilized, the 5′ J_α_ RSSs in the mammalian *Tcra* locus are generally of poor quality, indicating that other parameters, such as epigenetic modifications, may promote the efficiency of V(D)J recombination at these gene segments. On the other hand, the poor quality of V_β_ RSSs was proposed to reinforce allelic exclusion of *Tcrb* by limiting RAG1/2 activity. In a previous study, *in vivo* replacement of a poor quality V_β_ RSS with a more active RSS dramatically increased utilization of the contiguous V_β_ gene and reduced allelic exclusion, demonstrating the strong effect of RSS quality on V(D)J recombination efficiency and AgR repertoires *in vivo*.^19^ RSS divergence may have been selected to enforce allelic exclusion, suggesting RSS efficacy is just one factor in RSS evolutionary conservation. This would necessitate empirical characterization of the RAG1/2 DNA sequence specificity to accurately predict RSS efficacy.

## Evaluation of RSS Quality Using a High-Throughput Approach

Cryo-EM and X-ray crystallographic studies on RAG:RSS complexes showed extensive base-specific contacts at the near-perfectly conserved heptamer positions 1–3 and fewer contacts at heptamer positions 4–7.^2,7,12,20^ The RSS heptamer undergoes extensive conformational changes in the RAG1/2 active site, including unwinding 180° to position the scissile phosphate near the catalytic residues. This conformational flexibility might necessitate the absence of extensive base-specific RAG:RSS interactions because a 180° heptamer rotation flips minor groove contacts into the major groove and vice versa. These observations led to a hypothesis that RAG1/2 DNA sequence specificity is determined by a combination of base-specific interactions and sequence-specific conformational flexibility.^21^

**Figure 4:**
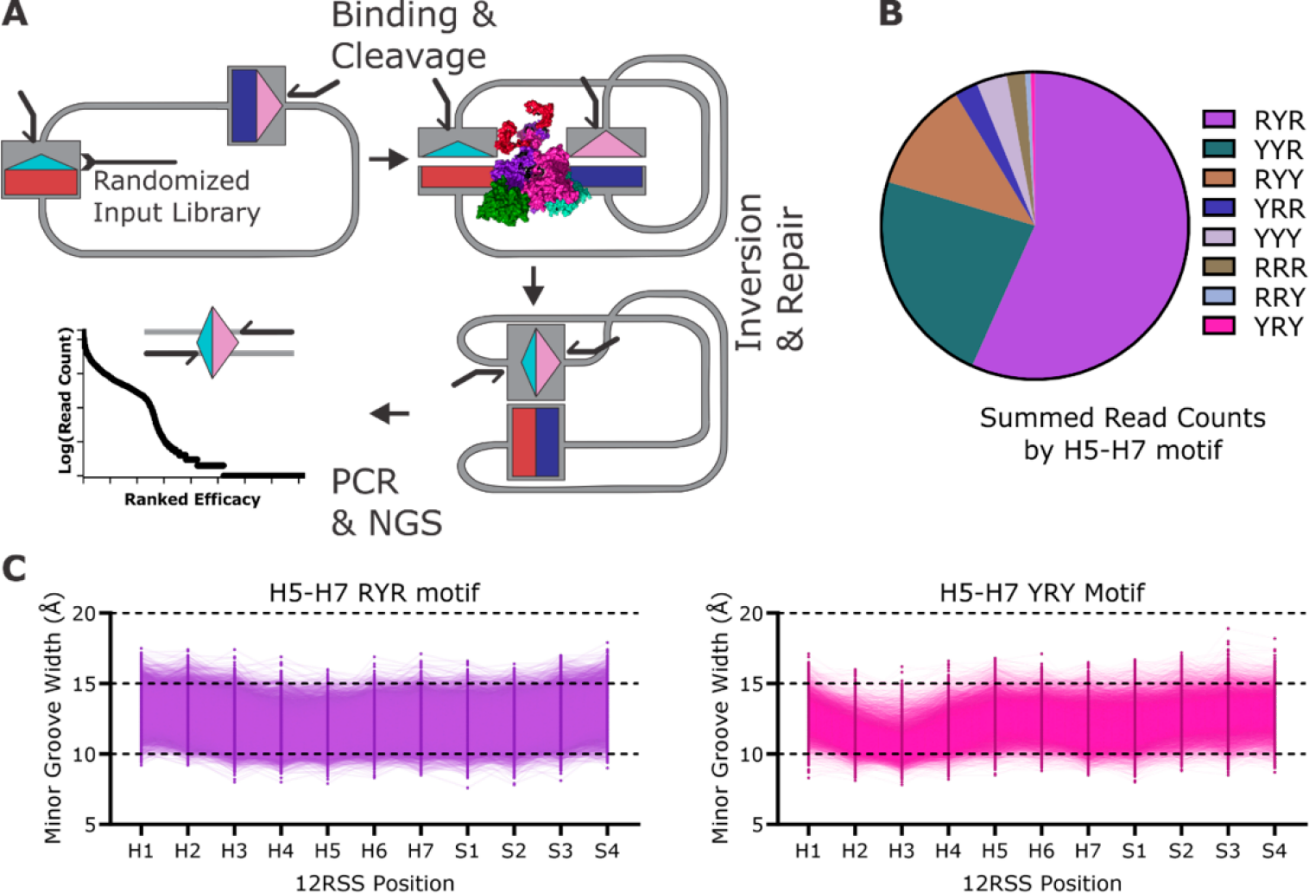
(A) A schematic workflow of the SARP-seq assay with a randomized 12-RSS sequence library inserted into the extrachromosomal V(D)J recombination plasmid substrate. PCR incompatible primer orientations are shown as bent black lines. RAG1/2 binds and cleaves the constant 23-RSS and the library of 12-RSSs with each sequence being recombined at different activity levels. Recombined plasmid products have a primer orientation that is compatible with PCR. Next-generation sequencing (NGS) of the recombined 12/23 RSS signal joints reveals differential 12-RSS utilizations. The relative recombination efficacy of each 12-RSS is determined by differential read count analysis. (B) Pie chart showing the relative efficacy of RSS heptamer motifs at positions 5–7, where R and Y are purine and pyrimidine, respectively. (C) The ranges of minor groove widths of 12-RSSs over the time scale of the MD simulations, which demonstrate minor groove width variability of the RYR in purple (consensus – CACA**ATG|**ATAC) and YRY in pink (anticonsensus – CACT**TAT|**GTAC) motifs at heptamer positions 5–7. The pie chart and plots shown in panels B and C, respectively, are representative data from Hoolehan et al.^21^

To determine how RAG1/2 recombines RSSs that are divergent in sequence through the conformationally flexible heptamer region, we revisited the question of RAG1/2 DNA sequence specificity for RSSs using a novel, high-throughput extrachromosomal V(D)J recombination assay called SARP-seq (selective amplification of recombination products and sequencing).^21^ SARP-seq increased the throughput of extrachromosomal recombination assays by orders of magnitude using randomized DNA substrates to simultaneously test recombination efficiency of thousands of RSSs (Figure 4A). The SARP-seq results showed a surprisingly large number of nonconsensus RSS heptamer sequences were recombined by the catalytically active core regions of RAG1/2.^21^ RSS heptamers were generally not favored at specific bases, save for T at heptamer position 6, but there was a clear preference for certain purine/pyrimidine (R/Y) motifs spanning heptamer positions 5–7. R/Y RSS motifs containing a YpR step at heptamer positions 6–7 were the only enriched R/Y motifs found in precise signal joints (Figure 4B), highlighting its importance for RAG1/2 cleavage compared with other RSS heptamer features.

YpR dinucleotides have low base-stacking energies and are conformationally flexible base-pair steps (see Table 2 in Marin-Gonzalez et al.^22^). YpR steps are deformable and can act as a flexible “hinge” in protein:DNA interactions. The preference for R/Y motifs rather than ACGT motifs might reflect sequence-specific DNA features that support conformational changes in the RAG1/2 active site because RAG-mediated cleavage does not require ATP as a source of free energy to drive a structural transition. In agreement with this model, molecular dynamics (MD) simulations of RSSs showed that DNA sequences preferred by RAG1/2 had dynamic structural features that may accommodate heptamer unwinding in the RAG1/2 active site.^21^ MD simulations revealed the CACNRYR motif had a more conformationally flexible minor groove than the opposite, anticonsensus CACNYRY motif (depicted in Figure 4C), and base-pair step parameters for RpY and YpR steps had opposite probability distributions (e.g., YpR steps preferred large roll angles while RpY steps preferred smaller roll angles). The most disfavored heptamer motif, CACNYRY, contains a YpR step at heptamer position 5–6 instead of 6–7. The flexible hinge may therefore be misaligned with respect to the scissile phosphate.

The interaction of RAG1/2 with the RSS involves simultaneously anchoring both the heptamer/coding flank and the nonamer, which is accomplished by enforcing extreme bends in the intervening spacer region. This configuration of the RSS likely induces a significant conformational strain on the DNA structure. The flexibility of the YpR base step and its propensity to adopt a non-B form structure are optimal for overcoming the energetic barrier for base unpairing through the RSS heptamer. The formation of specific interactions between RAG and the DNA melted form of the CAC sequence at the 5′ end of the heptamer would then function to stabilize the unwound form of the heptamer, which is prone for DNA cleavage at the scissile phosphate. RSS heptamers that do not contain the preferred YpR motif at base positions 6–7 would have a decreased ability to facilitate the base unpairing conformational change, and as mentioned above, these substrates may be misaligned such that the scissile bond is not stably positioned in the active site. It is also possible that the cRSSs that do not contain discernible heptamers or nonamers are able to be cleaved due to other mechanisms that lead to unpaired bases, thus bypassing the necessity for heptamer-related base unpairing prior to DNA cleavage.^23^

Because SARP-seq analyzed the complete V(D)J recombination reaction, *in vitro* biochemical studies on RAG1/2 nicking specificity will help determine whether some RSS motifs inhibit specific steps, such as nicking or hairpin formation. Future studies using this method, as well as additional structural studies and sm studies, will be important to fully understand the molecular basis for RAG activity in variant RSSs.

## Chromatin Landscape

Factors other than DNA sequence specificity affect the probability that RAG1/2 binds and cleaves at any particular RSS, such as the surrounding chromatin environment. However, it has been difficult to separate the contribution of the DNA sequence from epigenetic effects on RSS efficacy.

The discovery of RAG2 recruitment to H3K4me3,^4,24,25^ which is enriched in transcriptionally active open chromatin, opened the door to modeling RAG1/2 specificity for AgR gene loci in terms of highly plastic, transcription-coupled epigenetic modifications. The RAG2:H3K4me3 interaction was further characterized as an allosteric modulator of RAG1/2 DNA cleavage activity by relieving autoinhibition by the RAG2 noncore acidic hinge domain.^26^ In addition to regulating RAG1/2 cleavage activity, the RAG2 C-terminal noncore region couples RAG1/2 expression to the cell cycle by restricting expression to the G1-phase.^27^ Restricting RAG1/2 expression to the G1-phase protects against genomic instability and tumorigenesis in vivo.^28^ V(D)J recombination intermediates must be repaired by NHEJ after RAG-mediated cleavage to avoid deleterious chromosomal translocation caused by homologous recombination repair or alternative repair of signal or coding ends. NHEJ machinery is upregulated during G1, but the presence of a homologous DNA template during the S- and G2-phases favors homologous recombination repair. Residue T490 in the RAG2 noncore region specifically regulates cell cycle control, but it also increases RAG2 association with H3K4me3 and nuclear localization.^29,30^ Residue T490 is proximal to the PHD; therefore, post-translational modifications of T490 might regulate RAG1/2 localization by altering the PHD orientation, but this has not yet been experimentally verified.

The advent of next-generation sequencing technology opened the door to new approaches to understand V(D)J recombinase specificity. Multiple independent laboratories used chromatin immunoprecipitation sequencing (ChIP-seq), high-throughput genome translocation sequencing (HTGTS), and END-seq, among other NGS-based methods, to study RAG1/2 targeting and recombination activity.^4,31,32^ The Corcoran laboratory developed the VDJ-seq method to directly detect immunoglobulin recombination frequencies for all gene segments.^18^

Major challenges exist toward predicting correlation between activity and chromatin landscape, which include further understanding the numerous dynamic changes to the epigenetic landscape imparted by V(D)J recombination. These dynamic changes include nucleosome repositioning, transcription, transcription-coupled epigenetic modifications, and locus reorganization. Successfully analyzing active RAG:RSS interactions as they occur in developing lymphocytes will be an additional challenge, given the transient nature of the active RAG-RSS interactions and their asynchronous occurrence in a given cell population.

In addition to genetic and epigenetic factors, three-dimensional chromatin architecture is another important mechanism of V(D)J recombination regulation.^33,34^ RAG1/2 cleavage requires 12- and 23-RSS pairing; thus, two distal pieces of DNA must be captured by the enzyme complex. Chromatin conformation capture studies have identified topologically associated domains (TADs) that interact more frequently than those predicted by passive diffusion models of chromatin dynamics. If chromatin chains passively diffused without constraint, the probability of intrachromosomal contacts would decline as a function of distance between the contact points, but TADs within AgR loci indicate more stable intrachromosomal contacts that may facilitate the pairing of distal RSSs. RSSs within the same TAD could be more likely to pair than RSSs in separate TADs because of their spatial proximity. In AgR loci primed for V(D)J recombination, TADs are often stabilized by promoter and enhancer interactions that are distinctly positioned throughout the AgR loci. Evidence also indicates that the distinctive chromatin structures at AgR loci are “sol–gel” chromatin environments, which may facilitate site-specific contacts between RSS pairs and the V(D)J recombination machinery at the sol–gel interface.^35^ Recent studies from the Alt laboratory propose topologically associated loops within the contracted locus are extruded through a cohesin ring, a cohesin:CTCF complex anchors the loop, and that RAG1/2 selectivity is mediated by scanning.^36^ In this model, RAG1/2 scans DNA as it is extruded through the cohesin ring. Chromatin looping could prevent extreme bias toward recombination of proximal gene segments by bringing distal RSS pairs closer together. Regardless of the precise molecular mechanism, RSS pairs must be spatially proximal to form a PC with RAG1/2, and after PC formation, RSS efficacy would dictate the probability of coupled cleavage.

## Future Outlook

A better understanding of V(D)J recombinase specificity is needed to understand how RSS variance affects AgR gene utilization and to predict whether specific oncogenic translocations are RAG-mediated. SARP-seq experiments focused only on the 12-RSS heptamer and bordering spacer region and were performed only in the context of a consensus 23-RSS. Future high-throughput experiments focusing on the RSS nonamer-spacer region and 12/23-RSS pairing will identify additional DNA sequence determinants governing V(D)J recombination and the production of antigen receptor repertoires. Additionally, future SARP-seq studies performed with full-length RAG1/2 proteins will determine which, if any, noncore catalytic domains affect DNA sequence specificity.

Previous structural studies of RAG1/2 complexed with DNA revealed the structural mechanism by which RAG1/2 cleaves DNA, but there is no published structure of RAG1/2 in complex with a nonconsensus RSS. The SARP-seq results are consistent with many prior observations of the RAG:RSS complex, including the absence of base-specific contacts at heptamer position 4, the requirement for DNA deformability, the presence of a base-specific contact at heptamer position 6, and opening of the minor groove. However, one can only speculate on how the RAG1/2 active site would accommodate a nonconsensus RSS. Current electron density maps and cryo-EM density maps are poorly resolved around the DNA bases, leading to conflicting reports on their orientation in the RAG1/2 active site.^7,12^ It is possible that some nonconsensus RSSs may inhibit RAG-mediated cleavage by hindering necessary structural transitions through specific side-chain interactions and/or through misalignment in the RAG1 active site of the PC. Solving structures of RAG1/2 in complex with nonconsensus RSSs would provide insight into how the RAG1/2 active site accommodates divergent sequences.

Off-target DNA cleavage by RAG1/2 has long been known to be a significant source of genomic instability in developing lymphocytes. Most often, RAG-mediated off-target cleavage is believed to occur at cRSSs, which are present throughout the genome. It is predicted that there is one cRSS per 500 bp for a total of >5 × 10^6^ cRSSs present in the human genome,^37^ although only a subset of the cRSSs would be accessible to RAG1/2 in developing lymphocytes. In mice, direct evidence for RAG1/2 cleavage at cRSSs has been shown using END-seq and HTGTS, where both methods detected DNA ends arising from RAG-mediated DNA double strand break formation.^31,32^ In murine pre-B cells, 107 and 202 off-target RAG1/2 cleavage sites at cRSSs were detected by HTGTS and END-seq, respectively, with 54 of the cRSS sites detected by both methods. While all of the cRSS sites contained a CAC sequence at the 5′ end of the heptamer, many contained no obvious nonamer sequence. Defining the basis for RAG cleavage at some cRSSs, but not others, is needed to understand the potential and possibly limitations of off-target recombination events. It may be that cRSSs show a substantial increase in efficacy in V(D)J recombination when in a more favorable chromatin environment. Thus, future studies are needed to show if changes in the chromatin environment can render cRSSs more efficacious for RAG-mediated cleavage and any ensuing increase in aberrant genomic rearrangements.

Taken together, transcription coupled epigenetic modifications such as H3K4me3 and nucleosome depletion, spatial organization of chromatin into topologically associated domains, cohesin-mediated DNA loop extrusion, and hard-coded RSSs serve as a multilayered mechanism enforcing V(D)J recombination fidelity. Untangling these differing mechanisms contributing to V(D)J recombination activity will provide insight into the generation of AgR repertoires and disease mechanisms arising from off-target recombination.
